# Increasing Knowledge, Skills, and Confidence Concerning Students’ Suicidality Through a Gatekeeper Workshop for School Staff

**DOI:** 10.3389/fpsyg.2018.01233

**Published:** 2018-07-20

**Authors:** Rebecca C. Brown, Joana Straub, Isabelle Bohnacker, Paul L. Plener

**Affiliations:** ^1^Department of Child and Adolescent Psychiatry and Psychotherapy, University Hospital Ulm, Ulm, Germany; ^2^Department of Child and Adolescent Psychiatry, Medical University of Vienna, Vienna, Austria

**Keywords:** suicidality, gatekeeper, prevention, school staff, workshop, training

## Abstract

**Introduction:** Around one-third of adolescents in Germany report a lifetime history of suicide ideation. School staff (e.g., teachers or school social workers) can serve as gatekeepers to identify adolescents at risk and transfer them to appropriate mental health professionals. The aim of this study was to evaluate a gatekeeper training for school staff.

**Methods:** A total of *N* = 603 school social workers, school psychologists, and teachers participated in one of 33 1.5-day workshops. Knowledge, attitudes, confidence in skills, and perceived knowledge were assessed at pre and post workshops and at 6-month follow-up (FU). Behavioral changes were assessed via self-report at FU.

**Results:** Knowledge, perceived knowledge, and confidence in own skills concerning suicidality increased significantly from pre- to post-assessment and was still significantly increased at 6-month FU. Attitudes toward suicidal adolescents were neutral to positive before the workshop and remained un-changed at FU. Overall, participants were very satisfied with the workshop. Although participants stated to be motivated to make behavioral changes at 6-month FU, they reported obstacles such as lack of resources and support from school administration.

**Discussion:** This 1.5-day gatekeeper workshop was effective in enhancing knowledge and confidence in school staff regarding suicidality. Future workshops would benefit from ongoing supervision and inclusion of school administration in order to facilitate long-term changes on a behavioral level.

## Introduction

According the World Health Organization (WHO), suicide is the second leading cause of death among adolescents and young adults worldwide (World Health Organization, 2018). Although suicide rates are considerably small among adolescents compared to adults, suicide attempts and suicidal ideation are frequent in this age group. Around one-third of students in 17 European countries reported having had suicidal ideation at least once (Kokkevi et al., 2012), with slightly higher rates in Germany of 36.4–39.4% in school-based populations (Plener et al., 2009; Donath et al., 2014). Suicide attempts are reported by around 7–9% of German high-school students (Plener et al., 2009; Brunner et al., 2014; Donath et al., 2014).

Gatekeeper programs as an approach to prevent adolescent suicides have gained popularity in recent years. Evaluation of gatekeeper programs consistently showed an increase in knowledge and confidence in school staff after attending gatekeeper trainings (for review: Katz et al., 2013; Robinson et al., 2013). One of the most commonly evaluated gatekeeper programs is *Question, Persuade, Refer* (QPR), a short (commonly 1.5–2 h) group-training catered to different types of gatekeepers (e.g., school staff and community workers). QPR has shown positive effects with regards to increasing knowledge and self-efficacy in gatekeepers concerning suicidal adolescents (e.g., Coleman and Del Quest, 2015; Hangartner et al., 2018; Litteken and Sale, 2018). However, changes in actual behavior in prevention staff (e.g., asking at-risk adolescents about suicidality) were not always significant (Wyman et al., 2008; Hangartner et al., 2018). With regard to changes in suicidal behaviors, in a large European study (Saving and Empowering Young Lives in Europe; SEYLE), on the rate of suicide attempts in adolescents did not change significantly after teaching QPR to teachers (Wasserman et al., 2015). When adding role-plays to standard QPR, Cross et al. (2011) were able to increase effect sizes of QPR by 0.44 of a standard deviation regarding observed behavioral changes in prevention staff at 3-month follow-up (FU). When comparing three gatekeeper trainings [QPR, RESPONSE (2-h school staff training), and the more extensive (2-day long) Suicide Interventions Skills Training (ASIST)], only participants of ASIST showed significant increases in asking at-risk youth about suicide following the training. The authors concluded that more extensive training, including role-plays and modeling, are crucial to changing behaviors in prevention staff (Coleman and Del Quest, 2015). These results are underlined by a recent study showing positive effects in behavior in prevention staff, but also knowledge and skills for Australian school staff when dealing with suicidal adolescents after having participated in the Skills-based Training on Risk Management (STORM), including role-plays and active skills learning (Robinson et al., 2016). In conclusion, Susanne Condron et al. (2015) showed that participants of longer gatekeeper trainings showed more behavioral changes in prevention staff as those participating in shorter trainings. However, to our knowledge, except for the SEYLE study (Wasserman et al., 2015), no study has so far assessed the effect of gatekeeper trainings for school staff on actual suicidal behaviors in adolescents.

Although gatekeeper interventions seem to be internationally applied in order to prevent adolescent suicidality, no such intervention had been established in Germany before this study on a larger scale. Based on results of previous research, a more extensive workshop (1.5 days) including role-plays and skills’ training was implemented for school staff in Germany in our project. Significant increases in knowledge, perceived knowledge, confidence, as well as a significant reduction of negative attitudes were expected from before to after the workshop. These effects were expected to remain significant at 6-month FU. Furthermore, significant behavioral changes were expected at 6-month FU.

## Materials and Methods

### Participants

Workshops were free of charge to all school psychologists, school social workers, and teachers in the state of Baden-Wuerttemberg, Germany. In Baden-Wuerttemberg, school social workers have been increasingly implemented at schools in the past 10 years. School psychologists are the contact person for (school-related) mental health issues for teachers, students, and parents. They work remotely and are usually responsible for a large number of schools. School psychologists also supervise and train “counseling teachers,” who are regular teachers taking on an extra training. Counseling teachers have their office at the schools and set aside a certain number of hours a week from their teaching duties to counseling. School social workers are usually responsible for one to three schools (depending on their size) and usually have their office directly at the school, but are employed by external organizations. School social workers have more extensive training in mental health issues compared to counseling teachers. Apart from school social workers, counseling teachers, and external school psychologists, no other school welfare staff like school nurses exist.

Workshops were not compulsory and were advertised on the projects’ homepage and by emailing schools in respective areas the workshops took place in. Participants had to actively enroll in the workshop. Usually, one or two participants per school enrolled in the workshop, although this was not regulated.

In total, *N* = 603 participants completed a voluntary pre–post assessment (each having participated in one of the workshops). Data of those *N* = 603 participants are presented in this paper. Of those participants, *N* = 136 (28%) completed a 6-month FU online assessment, to which all participants were invited via a letter and email. Participants who completed the FU assessment did not differ significantly from those who did not, with regard to gender, profession, years of professional experience, satisfaction with the workshop, or any of the other measures at post assessment (*p* > 0.05 for all variables).

The majority of participants were school social workers (59.5%), 25.3% were teachers, 6.6% were school psychologists, and 7.6% identified as “other” (e.g., priests teaching religion in schools, or social workers working part-time at a school and part-time a youth-welfare institutions). Most participants were female (79.5%), had more than 1 year of professional experience (91.4%), and had been in contact with a student presenting with suicidality (71.3%) at least once.

### Workshop

In total, 33 workshops were delivered in different places around Baden-Wuerttemberg, Germany, between October 2014 and January 2018. On average, one workshop comprised 17 participants (min = 9, max = 32). The 1.5-day workshop was conducted by at least two presenters taking turns (one child and adolescent psychiatrist and three psychologists, all of which were licensed child and adolescent cognitive-behavioral therapists). All presenters had clinical experience in working with adolescents presenting with non-suicidal self-injury (NSSI) and suicidality.

The content of the workshop was adapted from workshops described by Robinson et al. (2008). Information presented in the workshop was derived from up-to-date scientific publications and clinical guidelines. On day 1 of the workshop, information on the epidemiology of suicidality and NSSI was given in a 1-h lecture. Afterward, the etiology of NSSI was conveyed by performing a stress test with the participants, video clips of adolescents with NSSI, and a 1-h lecture. In the afternoon, participants practiced in video-assisted role-plays on how to react to a student with NSSI. As an evidence-based method to enhance motivation for therapy in adolescents with self-harming behaviors, the method “Therapeutic Assessment” (Ougrin et al., 2009) was presented to participants in a 2-h session including a video clip and role-play. At the end of the day, basics of the stress tolerance skills training were presented and participants were able to try out skills themselves. On day 2, participants learned about the epidemiology and risk factors of suicidality, conveyed by a lecture, video clips, and self-directed learning. Afterward, participants practiced how to ask a student about suicidal thoughts in role-plays. As a last module, legal topics (e.g., when and how to involve parents or school administration) and worst case scenarios (e.g., when and how to call the police) were discussed, illustrated by case-reports and own experiences of participants. There was a clear separation of NSSI and suicidality throughout the workshop. Differences and similarities of both behaviors were stated, where applicable.

Although the workshop targeted both NSSI and suicidal behaviors, only results regarding suicidal behaviors are presented in this manuscript. For further details regarding results concerning NSSI and further details on contents of the workshop, see Groschwitz et al. (2017).

### Measures

Participants completed questionnaires directly before (pre) and after (post) the workshop. They were also invited to participate in an online FU evaluation 6 months after the workshop. Measures for evaluation were derived from Kirkpatrick and Kirkpatrick’s first three levels of their model for evaluation (*reaction, learning*, and *behavior*; Kirkpatrick and Kirkpatrick, 2006). *Reaction* was measured by participants rating their satisfaction with the workshop (29 items, Cronbach’s alpha = 0.90). *Learning* is divided into the areas of knowledge (16 items, multiple-choice questions), perceived knowledge (eight items, Cronbach’s alpha = 0.90), and confidence in own skills (eight items, Cronbach’s alpha = 0.91) in Kirkpatrick and Kirkpatrick’s model. At FU, participants indicated if they confident to identify suicidal adolescents or refer them to professional help and if they felt comfortable with asking a student about suicidality (I feel confident/comfortable vs. I do not feel confident/comfortable) and whether their handling of situations involving students with suicidal behavior had changed in the 6 months after the workshop on a day-to-day basis (five-point Likert-scale), and on how well they were able to integrate their knowledge on a school level (five-point Likert-scale; *Behavior*). In addition to this model of evaluation, changes in attitudes toward adolescents with suicidality (seven items, Chronbach’s alpha = 0.82) were assessed, since this has shown to be relevant in previous studies (i.e., Heath et al., 2011). All items (except for the knowledge test which consisted of multiple-choice questions) were rated on a five-point Likert scale (1 = fully agree to 5 = do not agree). For the knowledge test, answers were coded as 1 = correct and 0 = incorrect. A mean of correct answers [all correct and incorrect answers added up and divided by number of items (16)] was calculated. For example, a total score of 1 indicated 100% correct answers, while a score of 0 indicated 0% correct answers. A more detailed description of the evaluation tool was published by Groschwitz et al. (2017).

### Statistical Analyses

Data were analyzed using the statistic software IBM SPSS Statistics, version 25. As participation in the FU assessment was rather low (28%), pre–post analyses (regarding knowledge, perceived knowledge, confidence in skills, as well as attitudes) were conducted for all *N* = 603 participants, including the between-subject variable “profession.” Differences between all three time-points (*N* = 132) were calculated using a repeated measures analysis of variance (ANOVA) across all three time-points. *Post hoc* *t*-tests were performed. For significant differences, effect sizes (Cohen’s *d, repeated measures*) were calculated.

### Ethics

The study was carried out in accordance with the Declaration of Helsinki and was approved by the institutional review board of the University of Ulm.

## Results

### Satisfaction With the Workshop

In general, participants were very satisfied with the workshop (*M* = 4.8, *SD* = 0.3; on a scale from 1 = not satisfied to 5 = very satisfied). They were most satisfied with the workshop leaders (*M* = 4.8, *SD* = 0.3) and the atmosphere (*M* = 4.8, *SD* = 0.4). Participants were also very satisfied with contents of the workshop (*M* = 4.6, *SD* = 0.3), time management (*M* = 4.6, *SD* = 0.4), as well as technical equipment (*M* = 4.5, *SD* = 0.6). Furthermore, 89.3% stated to definitely and 10.2% stated to most likely recommend the workshop to colleagues.

### Pre–post Changes

Knowledge, perceived knowledge, and confidence in skills increased significantly (*p* < 0.001) from before to after the workshop. Furthermore, negative attitudes toward suicidal students decreased significantly (for details, see **Table 1**).

**Table 1:** Data of pre–post assessment.

	*M*_pre_ (*SD*)	*M*_post_ (*SD*)	*T*	*d*_repeated measures_
Knowledge	0.32 (0.18)	0.68 (0.17)	−37.6^∗∗∗^	1.6
Perceived knowledge	2.9 (0.8)	3.9 (0.5)	−26.4^∗∗∗^	0.9
Confidence in skills	3.0 (0.9)	4.1 (0.5)	−27.5^∗∗∗^	0.9
Attitudes	1.8 (0.7)	1.4 (0.5)	11.4^∗∗∗^	−0.5

*^∗∗∗^p < 0.001.*

### Differences Between Professions Regarding Changes From Pre- To Post-workshop

**FIGURE 1:**
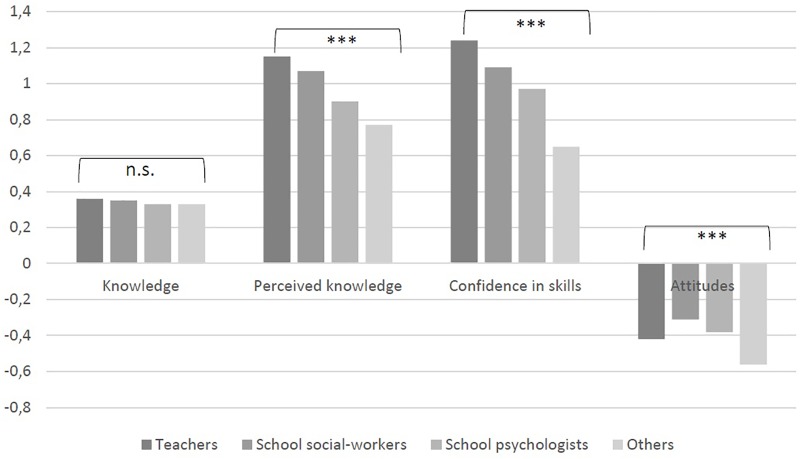
Changes in knowledge, perceived knowledge, confidence in skills, and attitudes from before to after the workshop by profession. ^∗∗∗^*p* < 0.001; n.s., not significant.

Between-subject effects regarding profession were calculated for changes pre- and post-workshop with regard to knowledge, perceived knowledge, confidence in skills, and attitudes (for details, see **Figure 1**). There were no significant differences with regard to increases in actual knowledge between professions (*F* = 0.12, *p* = 0.94). Increases in perceived knowledge and confidence in own skills differed between professions (*F* = 3.73, *p* < 0.05 and *F* = 4.21, *p* < 0.05, respectively), with teachers reporting largest increases, and “other” professions reporting smallest increases, followed by school psychologists. Decreases in negative attitudes also differed significantly between professions (*F* = 4.2, *p* < 0.05), with other professions and teachers showing largest decreases in negative attitudes, while school social-workers showed smallest decreases (for details, see **Figure 1**).

### Follow-Up Assessment

Repeated measures ANOVAs showed significant differences between all three time-points (pre, post, and FU) for all measures (knowledge: *F* = 161.4, *p* < 0.001; perceived knowledge: *F* = 240.3, *p* < 0.001; confidence in skills: *F* = 199.2, *p* < 0.001; and attitudes: *F* = 11.4, *p* < 0.001).

**FIGURE 2:**
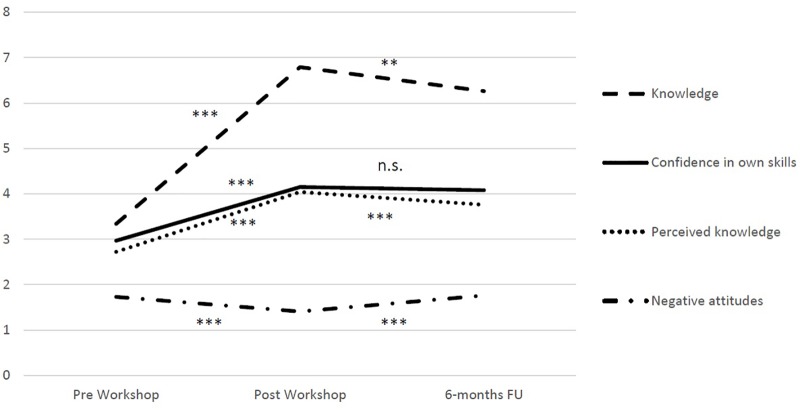
Changes in knowledge, confidence in skills, perceived knowledge, and negative attitudes from pre to follow-up (FU). ^∗∗∗^*p* < 0.001 and ^∗∗^*p* < 0.01; n.s., not significant; 6-months-Fu, follow-up at 6 months.

*Post hoc* *t*-tests revealed significant increases in knowledge from pre to FU (*T* = −12.6, *p* < 0.001, *d* = 1.9), but also significant decreases in knowledge from post to FU (*T* = 2.6, *p* < 0.05, *d* = −0.03). Perceived knowledge increased from pre to FU (*T* = −14.6, *p* < 0.001, *d* = 1.1) and also decreased significantly from post to FU (*T* = 4.9, *p* < 0.001, *d* = −0.5). Confidence in skills increased significantly from pre to FU (*T* = −15.0, *p* < 0.001, 1.5) and did not decrease significantly from post to FU (*T* = 1.2, *p* = 0.23). Attitudes were the same at pre and FU (*T* = 0.00, *p* = 1.0), which was due to a significant increase in negative attitudes from post to FU (*T* = −2.5, *p* < 0.001, *d* = 0.5; for details, see **Figure 2**).

At FU, 81.5% of participants stated they felt confident to identify students at risk, 92.6% felt comfortable asking a student about suicidality, and 85.9% felt confident in referring a student to professional mental health care.

Furthermore, 55.2% of all participants stated to have found the contents very useful for their daily work and 34.3% stated to have found them useful. The same was true for motivation to implement contents of the workshop, with 54.8% stating to be very motivated and 37.8% stating to be motivated. However, only 23.5% stated to have transferred those skills very successfully into their daily work, while 44.1% stated to have done so successfully, and 27.9% stated to have done so a little. Furthermore, 15.7% stated to have changed their behavior with regard to suicidal students very much, while 39.6% stated to have done so moderately, and 34.3% stated to have done so a little. Changes on a school level were only observed by 13.4% of participants. Only around a third of participants had shared the contents of the workshop with school administration (37.0%), or were working with school administration to change the way the school handled suicidal students (30.2%). In the same line, only 33.1% stated that school administration was open for new concepts when dealing with suicidal adolescents, and only 29.2% reported sufficient timely resources to make changes at a school level.

## Discussion

Results of this study showed that a 1.5-day gatekeeper training for school staff can significantly change knowledge, perceived knowledge, and confidence in skills regarding student suicidality, with effects still being significant after 6 months. While negative attitudes toward suicidal students were reduced significantly directly after the workshop, attitudes returned to their original level at FU. Differential effects by profession of gatekeepers were found. Behavioral changes (assessed by self-report) showed moderate effects. Main obstacles for behavioral changes were identified by participants as a lack of support from school administration and timely resources.

In line with previous studies evaluating gatekeeper trainings for school staff, knowledge, perceived knowledge, and confidence in own skills were significantly increased by this workshop (Wyman et al., 2008; Hangartner et al., 2018). These changes are very important, as school staff are recognized as important gatekeepers for suicide prevention by the WHO (World Health Organization, 2012/2018). In a study asking adolescents about their help-seeking behaviors, teachers were named a source of help in the case of self-harm before mental health professionals or general doctors (Fortune et al., 2008). Factual knowledge, and especially perceived knowledge, can increase one’s confidence in their own skills and can in turn enable a person to react and act appropriately in difficult situations.

Different from other studies (e.g., Wyman et al., 2008), negative attitudes toward suicidal adolescents were not reduced significantly at FU, even though they had significantly reduced directly after the workshop. One reason might be that attitudes were already quite positive before the workshop (1.8 on a scale from 1 = not at all negative to 5 = very negative), which may have resulted in a ceiling effect. As participants had to enroll actively in the workshop, it is likely that mainly participants who did not hold negative attitudes toward those adolescents enrolled in the workshop, as they were interested in providing better care.

On the other hand, despite negative attitudes returning to their original level at 6-month FU, perceived knowledge and actual knowledge also decreased significantly from post to FU (although still significantly higher at FU than before the workshop). A lack of sustainability of effects of gatekeeper suicide prevention trainings has been found in several studies. Results of a qualitative study performing in-depth interviews and focus groups with gatekeepers suggest that (Shtivelband et al., 2015) implementing a social network where participants can stay in touch with other participants of the workshop and exchange experiences, continued learning (e.g., via additional online material), community outreach programs after the workshop, and reminders and ongoing communication might enhance sustainability of increased knowledge, and confidence in own skills.

Significant differences were found between the different professions (teachers, school social-workers, school psychologists, and “others”) this training was delivered to. Presumably, teachers benefit most from the training, while other professions (who the workshop was not catered to specifically) and school psychologists had the least benefit. These results suggest to be specific in who to deliver the workshop to (mainly school social workers and teachers) and adjust contents to school psychologists (who usually have higher previous knowledge and skills) and other professions (who predominantly might work in different settings than schools).

Findings at 6-month FU have to be interpreted with care, due to the high drop-out rate. Even though participants who completed FU did not differ significantly from those who did not on any socio-demographic variables or behavioral levels or satisfaction with the workshop at post-assessment, some possible biases might exist (e.g., participants who completed the FU assessment may have been involved in the topic on a more regular basis during the 6-month FU period or had more timely resources available to them).

One important effect at 6-month follow up was that almost all participants felt confident in identifying students at risk and in asking a student about suicidality. This is particularly important as a study by Carlton and Deane (2000) showed that students aged 14–18 years reported lower help-seeking intentions with increasing suicidal ideation. Therefore, it is important for gatekeepers to feel confident in actively identifying and consequently approaching students at potential risk for suicide.

While motivation was high to use contents of the workshop, the majority of participants had not transferred skills learned in the workshop into their daily work. One reason for this outcome might be that most participants (especially teachers) would not have to deal with suicidal adolescents on a day-to-day basis and may therefore not have used skills learned in the workshop frequently. However, this enhances the notion of continuous training after workshop completion (as suggested by Shtivelband et al., 2015), as knowledge and skills might decrease continuously over time, if not reinforced and practiced.

Another important obstacle of implementation of newly acquired skills, and especially of being able to spread knowledge and skills to colleagues at the school, seemed to be the lack of support from school administration and timely resources. It may therefore be viable for gatekeeper trainings (especially in the school system) to include and inform administration, in order to achieve highest possible support for participants. However, this might not always be feasible, as schools have to cope with many problematic issues (e.g., bullying or drug-abuse), and may not always regard suicidality as the most important issue.

### Limitations

The major limitation of this study is that behavioral changes were only assessed in self-report and no objective measures (e.g., rate of suicide attempts) were assessed. As almost all participants worked at different schools, however, it was not feasible to assess those measures due to time and financial restraints. Furthermore, school authorities in Germany are increasingly reluctant to allow studies on suicidality in schools, although evidence clearly shows that such studies do not provoke suicidality in students or cause other harm (Gould et al., 2005; Lloyd-Richardson et al., 2015). Another limitation is the drop-out rate at FU. However, participants at FU did not differ significantly from those who did not complete FU on any measure. There may still have been a bias of those participants who were still particularly engaged in subjects of the workshop at FU, to respond to the invitation. Therefore, results at FU have to be interpreted with care. Additionally, participation in the workshop was voluntary, which possibly favored motivated and interested school staff to participate and may have biased pre–post and FU results.

## Conclusion

Participants of this 1.5-day gatekeeper workshop for school staff were highly satisfied and showed significant benefits with regard to knowledge, perceived knowledge, and confidence in skills. Most participants felt confident to identify a student at risk and approach them to ask about possible suicidal thoughts and behaviors at 6-month FU. As school staff are important gatekeepers in the prevention of adolescent suicide, gatekeeper workshops are recommended. However, future studies need to assess objective outcomes (like rates of suicidal ideation or suicide thoughts) in order to make broader recommendations. Future workshops should be catered to individual professions (i.e., separate workshops for teachers and school psychologists), in order to provide best possible benefits. Furthermore, participants would benefit from ongoing support after the workshop in order to sustain benefits in the long term.

## Author Contributions

RB, JS, and PP were involved in the design of the study. RB analyzed the data, performed statistical analyses, and drafted the manuscript. All authors were involved in obtaining data and delivering training, critically reviewed the manuscript, and gave their consent to the final version.

## Conflict of Interest Statement

PP has received research funding from the Federal Institute for Drugs and Medical Devices (BfArM), BMBF (Federal Ministry of Education and Research), VW Foundation, Baden-Wuerttemberg Stiftung, Lundbeck, Servier. He received speaker’s honorarium form Shire. He holds no stocks of pharmaceutical companies. The remaining authors declare that the research was conducted in the absence of any commercial or financial relationships that could be construed as a potential conflict of interest.
